# A Framework for Planning and Execution of Drone Swarm Missions in a Hostile Environment

**DOI:** 10.3390/s21124150

**Published:** 2021-06-17

**Authors:** Barbara Siemiatkowska, Wojciech Stecz

**Affiliations:** 1 Institute of Automatic Control and Robotics, Warsaw University of Technology, 02-525 Warsaw, Poland; 2 Faculty of Cybernetics, Military University of Technology, 00-908 Warsaw, Poland; wojciech.stecz@wat.edu.pl

**Keywords:** mission planning, UAV swarms, object detection, CNN, SAR, EO

## Abstract

This article presents a framework for planning a drone swarm mission in a hostile environment. Elements of the planning framework are discussed in detail, including methods of planning routes for drone swarms using mixed integer linear programming (MILP) and methods of detecting potentially dangerous objects using EO/IR camera images and synthetic aperture radar (SAR). Methods of detecting objects in the field are used in the mission planning process to re-plan the swarm’s flight paths. The route planning model is discussed using the example of drone formations managed by one UAV that communicates through another UAV with the ground control station (GCS). This article presents practical examples of using algorithms for detecting dangerous objects for re-planning of swarm routes. A novelty in the work is the development of these algorithms in such a way that they can be implemented on mobile computers used by UAVs and integrated with MILP tasks. The methods of detection and classification of objects in real time by UAVs equipped with SAR and EO/IR are presented. Different sensors require different methods to detect objects. In the case of infrared or optoelectronic sensors, a convolutional neural network is used. For SAR images, a rule-based system is applied. The experimental results confirm that the stream of images can be analyzed in real-time.

## 1. Introduction

Planning and supervising the course of missions carried out by drone swarms in a dangerous environment requires taking into account the need to re-plan the mission, which usually means at least the need to change the planned flight route. Considering the fact that the drone swarm performs tasks related to the recognition of one or more objects in the field, changing the drone flight plans also requires the definition of a new payload work plan. New configuration parameters must be defined for each sensor with which a swarm’s UAV is equipped so that the collected reconnaissance data are of the best quality.

The process of changing the swarm’s flight plan may be initiated automatically or semi-automatically by algorithms for detection of dangerous objects located on the swarm’s flight route. For the process to be efficient, each UAV should be equipped with algorithms for automatic detection of objects in photos and/or SAR scans. After detecting a threatening object, the UAV may try to determine a new flight route.

This article presents the methods of planning and correcting the route plan based on the data provided by algorithms for automatic detection of dangerous objects.

The term UAV swarm denotes a group of drones performing a mission where all tasks are linked. Each drone is assigned a task to be performed. Some drones can be used to recognize or identify objects at close range using EO/IR devices. Others can use synthetic aperture radar (SAR) to recognize objects from a distance of several kilometers.

This article is a continuation of one of the authors’ work [1], which describes how to plan a mission for a group of drones performing reconnaissance tasks, but not cooperating with each other.

Using the categorization of drone swarms described in [2], it can be said that the article presents a model of operation of a drone swarm that uses EO/IR to identify targets at close range and SAR from a distance of more than 8 kilometers. All drones communicate with each other, but only one of the drones is equipped with a radio link that allows communication with the ground control station (GCS). This drone is used as an element for transmitting control messages from the GCS to any of the drones. This model of operation is widely used in military reconnaissance drones. The drone that connects the GCS to the swarm is called the information node.

In order to make the presented model more flexible, it is assumed that the UAV retranslator is equipped with a SAR and conducts additional reconnaissance of the area itself. In the presented model, it is also assumed that the drone swarm may consist of several information hubs, each of which manages its own group of drones. To the best of our knowledge, there are no models in the literature that present such a configuration of the swarm’s operation. The form of the optimization task and its connection with the object detection algorithms proposed in this article is the most important research achievement.

The rest of this article is organized as follows. In Section 2, the reader is introduced to the current state of modeling the operation of drone swarms that are equipped with threat-detection algorithms based on the analysis of collected photos and scans. This part also presents the works that formed that basis for the model in question. Section 3 presents the optimization model used for planning a swarm mission. The model of the network representing the area used to determine the swarm’s flight routes is described (Section 3.2). Then, the task of optimizing the route plan on the constructed network of connections is presented (Section 3.3, Section 3.4, Section 3.5). Task constraints are divided into groups. The first group includes the constraints used to model drone missions that do not need to work together. The second group shows the constraints of network flow behavior in routed networks. The third group presents constraints on a swarm of drones in which one UAV coordinates the work of the others. Several variants of the objective function are presented and used in the experiments (Section 3.6). In Section 3.7, a route replanning scheme is presented. The next section presents the convolutional neural networks (CNNs) for object detection with IR and OE cameras (Section 3.8). In Section 3.9, SAR image preprocessing, segmentation, region analysis and classification algorithms are described. Section 4 presents the most important results of the conducted experiments. Section 5 presents the conclusions of this work.

## 2. Related Work

Planning of drone missions, an important part of which is to determine the drone flight route and how to use the payload, is of particular importance when managing a swarm mission consisting of many cooperating unmanned platforms. Depending on the class of the platform, the sensors installed may have different purposes. In small platforms, they only support navigation and are used for detection of obstacles in the field at short distances from the UAV. In this case, the flight planning subsystem dynamically builds a new flight trajectory. Determining such a trajectory corresponds to solving a local optimization problem that takes into account the obstacles to be avoided. A good example of this type of method is the method shown by Wen et al. [3]. In this method, a dynamic domain rapidly-exploring random tree is combined with the motion planning method when searching local paths under threats and uncertainties that may be met during a mission execution.

This article uses a different assumption. Due to the fact that medium-altitude long-endurance (MALE) class platforms and tactical class platforms are able to recognize targets and detect threats from a distance exceeding several kilometers, it can be assumed that the route plan prepared before the mission can be recalculated or, in fact, corrected only for a part of the designated route. Therefore, numerical methods based on vehicle route planning with time windows (VRPTW) tasks can be used, which provide more accurate solutions at the expense of finding them. A new plan can be designated at the ground control station and sent to the UAV as a new task. However, the platform has sufficient hardware capabilities on-board to solve such a task.

**Figure 1:**
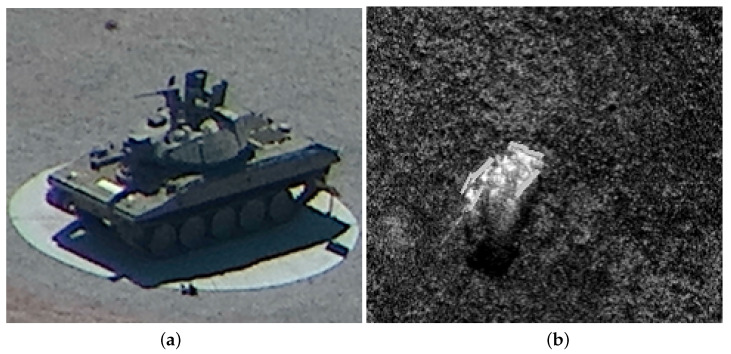
Tank images taken with (**a**) EO camera; (**b**) SAR.

This article describes the planning of drone swarm missions for drones that recognize objects in the field. In the article, a swarm of drones is understood as a group of unmanned aerial platforms carrying out a common mission. In such a mission, each of the drones is assigned a task. Some drones can be used to identify targets at close range with EO/IR systems. Others use SAR radars to identify the target from a distance of several kilometers. The detection of dangerous objects, such as tanks, convoys or trucks, is an essential part of the drone mission. Object detection is utilized in many applications. However, when compared to classic methods, item detection in aerial images has many different challenges. First of all, drones are equipped with different types of sensors: CCD cameras, infrared cameras, optoelectronic sensors and SARs. The same object looks quite different in photographic and SAR images. Aerial images are also noisy and have a smaller scale. Figure 1 presents images of tanks taken with an optoelectronic sensor (EO) (Figure 1a) and SAR (Figure 1b).

In the literature related to the planning of drone swarm missions, drone swarms are often categorized according to the way the swarm is moving, or the rules of communication between individual unmanned platforms. The method of using the payload during target recognition is also taken into account. Based on the classification presented in Boskovitz’s [4] research, the problem of UAV swarm intelligence can be decomposed into five layers: the mission decision-making layer, the planning layer, the control and communication layer and the application layer.

In the decision-making layer, mission plans are determined based on tasks to be performed, which are assigned specific priorities. The planning layer defines the details of the mission, such as determining the flight path of a swarm of drones. In the control layer, the implementation of the swarm’s mission is supervised, including the method of avoiding obstacles or the adopted formation of the swarm’s flight. In the communication layer, it is planned how to ensure the connection between drones, the scope of information exchanged or the fusion of data obtained by drones. The application layer determines the environment in which the drone swarm is used. A detailed description of the classification of drone swarm mission management algorithms is given in the study [5].

The framework for planning and execution of a drone swarm mission in a hostile environment presented in this article is based on components from two layers: the planning layer and the application layer. In the planning layer, the swarm’s flight paths are determined. In the application layer, methods for the detection of objects of various types are implemented. The results of these algorithms are then used to re-plan the missions.

The basic method of modeling the flight paths of a swarm of drones is to prepare the model in the form of a MILP class task. VRPTW models are usually built, which describe the movement of drones that visit the indicated places at given time intervals. Proper development of the model guarantees obtaining the optimal solution, provided that one exists. Unfortunately, it usually takes a long time to find such a solution and, therefore, heuristic algorithms are usually used.

The description of even some of the most important articles constituting the basis of route planning algorithms on the directed network exceeds the scope of this article. The interested reader is referred to the very deep overview of algorithms given by Zhou [5].

Nevertheless, among many works in the field of route planning, the following algorithms can be mentioned: ant colony optimization [6], particle swarm optimization [7], the bee colony algorithm [8], the multi-swarm fruit fly optimization algorithm [9] and the firefly algorithm [10]. Genetic algorithms are also used for route planning. Xin et al. [11] proposed a modification to the algorithm that increases the efficiency of the process of generating new populations. Arc routing problems are described by Liu et al. [12], who discussed the problem of capacitive arc routing that minimizes overall travel costs. Another work by Chow [13] investigated a drone routing problem that required multiple visits to the arcs.

The model presented in this article plans drone swarm missions in guided networks consisting of several groups of constraints. The model is based on the source model presented in Stecz [1,14]. The article shows the model’s extensions that allow an analyst to plan a swarm mission. The first group consists of constraints on the behavior of the flow in networks. The second group consists of constraints specific to the UAV performing reconnaissance tasks. The group takes into account the requirements for sensors. The last group consists of constraints related to the planning of swarm operations, in which individual UAVs exchange information with each other.

A classic system of object detection consists of three stages: segmentation, feature extraction and classification. During the segmentation process [15], the regions that contain the object of interest are extracted. The most popular are thresholding methods, multi-Otsu algorithms or sliding-window techniques [16]. In each extracted region, the features are extracted. Usually, scale-invariant feature transform (SIFT) [17], the histogram of oriented gradients (HoG) [18] and Haar-like [18] features are used during classification. Support vector machine (SVM), naive Bayes (NB) and random forest (RF) are the most efficient and robust classifiers [19]. Recently, convolutional neural networks (CNNs) in image processing and object detection have made tremendous progress [20]. They usually require one stage in the process of detection (e.g., RetinaNet [21] and You Only Look Once net (YOLO) [22]), or a two-stage detection (e.g., Fast R-CNN [23]). CNNs have millions of parameters, so they require large datasets. Public, well-annotated databases of different types of objects are available (ImageNet [24], CitySpace [25]). However, they do not contain items relevant to the military application.

## 3. Swarm Mission Planning

### 3.1. A Framework for Mission Planning

**Figure 2:**
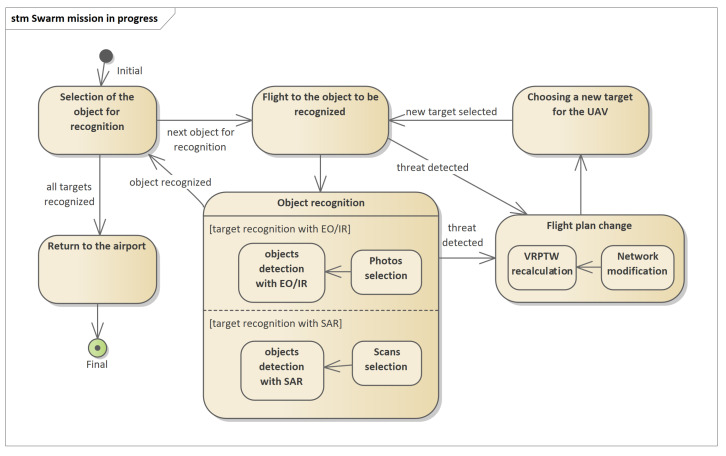
Schematic diagram of the object recognition process with the use of available sensors (EO/IR and SAR) in the form of a state machine in UML notation. If a threat is detected, the flight plan change process is triggered.

The mission planning process described in this article concerns determining the routes of swarm flights to recognized objects, the location of which is known before starting the planning. This is the most common object recognition task. The reconnaissance mission process is shown in Figure 2. To start the mission, a mission plan should be sent to the UAV, as described briefly in [1]. The mission plan includes the route of the UAV and a set of objects to be recognized. As part of the recognition of the object, the following activities are performed: selection of the sensor type, setting of the sensor operating parameters, carrying out the recognition using the sensor, recording the data from the recognition and data processing.

Usually, the reconnaissance is performed on-line by payload operators (except for missions carried out in the autonomous mode without contact with the GCS). However, this is not the only way to conduct reconnaissance. Another method of operation is the reconnaissance carried out by the UAV operating in an autonomous mode, when data from selected sensors is first collected and then sent afterwards, or sent immediately but only when a particularly dangerous object is detected.

In both of the above cases, UAV management systems must implement procedures that allow for automatic processing of collected data in order to identify direct threats to flying platforms. For platforms operating only in GCS control mode, such procedures are GCS software components. In the case of platforms operating as standalone, threat identification packages must be implemented on these platforms. This article presents the concept of building a system for an autonomous platform that uses dedicated software to identify threats based on data collected from the EO/IR and SAR.

Figure 2 shows a diagram of the mission execution process by the UAV in an autonomous mode. While recognizing objects with the use of available sensors (function object recognition), the UAV automatically identifies and classifies the objects found. Based on this, the UAV can determine whether they constitute a direct threat to the air platform. If such a threat is identified, the flight plan change function is triggered.

Each UAV is described by the following parameters: the maximum possible flight time of the UAV (τ), the type of UAV (*q*) and the set of sensor types with which the UAV is equipped $\left\{\omega_{s}\right\}$, where *s* is a sensor index.

The presented model describes the planning of missions of two types of drone. The first type includes drones equipped with EO/IR sensors. All of them can recognize objects at close range. The second group includes UAVs that act as information nodes connecting the GCS with the first group of drones. UAVs belonging to the second group may be equipped with sensors enabling recognition from a greater distance (most often SAR).

### 3.2. Terrain Model for Mission Planning

The representation of the terrain model on which drone swarm missions are planned depends on the adopted method of modeling the swarm optimization task. This article uses the tasks planned on the network of connections, which requires the preparation of the network described in [1,14].

The network used in terrain modeling is denoted as S=<V,E> where V models the set of vertices, |V|=V, and E models the set of edges, |E|=E.

Each vertex of the network S is described by the coordinate vector $<{\left(x,y,z\right)}_{i}>$, i=1…V. The vertices modeling the places where the reconnaissance task begins may be additionally described with such parameters as:

$p_{i}$—the profit gained when the vertex is recognized by the UAV,
$T_{i}^{I}$—the recognition time needed for the UAV to complete the task,
$e_{i}$—the earliest possible date and time of object recognition,
$d_{i}$—the latest possible end date and time for a scheduled object recognition.

Each arc of S is described by adjacent vertices (i,j)∈E.

The network edges model the route segments that can be divided into two groups. The first group includes the edges on which the UAV performs reconnaissance. Edges are assigned the highest priority. In the optimization task, the priority corresponds to the profit from planning a flight on a given route segment. The process of determining the position of the points in the field that are modeled with the highest priority vertices and edges is very complicated. The position of the vertices and edges depends on a few basic things: the anticipated dislocation of the recognition target, the terrain and the sensor that can be used for recognition. This is described in the article [1]. The object identification algorithms described in this article are crucial in this case. The maximum profit value is predefined. The second group includes the edges on which the UAV has to fly in order to reach the reconnaissance segment of the route. Depending on the task, the values of the priorities (profits) may be changed. This depends on the individual assessment of the planner. This is the normal operating procedure for planning a UAV mission. The vertices of the network representing points in the field can have different meanings. In the case of segments where the UAV conducts reconnaissance, the vertices model the places where the reconnaissance begins and ends. In the vicinity of such places, other vertices modeling the places where the sensors are calibrated are often determined. There may also be places where the UAV starts sending photos or SAR scans if the mission plan specifies that the UAV cannot send data during reconnaissance. The set of network vertices may include vertices and edges that model the route segments of the flight that are safe for UAVs. An example of the method of determining such segments is shown in [1], where the method of generating networks in the area with dangerous objects is illustrated.

The network model presented in this article has been extended in relation to the base model used in the works [1,14]. The article takes into account that the vertices of the network can be located at three different altitudes, which allows for efficient recognition of objects of various types. By default, the drones belonging to the swarm fly at predefined operational levels that minimize energy consumption. In the presented article, this assumption has been modified.

### 3.3. Constraints for UAVs without Cooperation

This part of the article presents the problem of swarm flight planning based on the formulation of the vehicle route planning with time windows (VRPTW) task in the form of mixed integer linear programming (MILP). Such a definition of the problem allows finding the optimal or close to optimal solution, when the analyst does not want to wait for the solver to complete the calculations. The present article introduces a number of new constraints related to the modeling of a swarm of drones. Some of the constraints presented in the articles [1,14] have been modified. These constraints will be discussed in detail in this article. Model variables are as follows:

$x_{ih}=1$ if the UAV h∈H visits the waypoint i∈V; 0 otherwise.
$y_{ijh}=1$ if the UAV h∈H visits the edge (i,j)∈E; 0 otherwise.
$t_{ih}$ describes the arrival time of the UAV h∈H to the waypoint i∈V.
$f_{ij\omega h}=1$ if the target assigned to the route segment $y_{ijh}$ is recognized with the sensor ω∈Ω by which the UAV h∈H is equipped; 0 otherwise.

The most important constraints defined in the swarm flight model are presented below. These constraints are similar when modeling the flight of single UAVs and swarms of drones. Some of them are described in [1]. In this article, some constraints have been modified for the needs of swarm flight modeling.

(1) $$\begin{array}{c} t_{jh}\geq t_{ih}+T_{ij}\cdot y_{ijh}-M\left(1-y_{ijh}\right)+x_{ih}\cdot T_{i}^{I},\\ \forall (i,j\in V:j\neq i,h\in H) \end{array}$$

(2) $$x_{ih}\cdot e_{ih}\leq t_{ih}\;,\;\forall \left(i\in V^{\prime }\subset V,h\in H\right)$$

(3) $$x_{ih}\cdot d_{ih}\geq t_{ih}\;,\;\forall \left(i\in V^{\prime }\subset V,h\in H\right)$$

(4) $$\begin{array}{c} x_{ih}-x_{jh}=0,\forall (i\in V:i\in \left\{k,l\right\},\\ j\in V:j\in \{m,n\},h\in H) \end{array}$$

(5) $$\sum_{i\in V:i\in \{k,m\},j\in V:j\in \{k,m\},i\neq j}y_{ijh}\leq \gamma ,\forall h\in H$$

(6) $$\sum_{i\in V:i\in \{l,n\},j\in V:j\in \{l,n\},i\neq j}y_{ijh}\leq \gamma ,\forall h\in H$$

(7) $$\begin{array}{c} \sum_{i,j\in V:i\neq j}T_{ijh}\cdot y_{ijh}+\sum_{i\in V}x_{ih}\cdot T_{i}^{I}\leq \tau_{h},\forall h\in H \end{array}$$

In optimization problems with time constraints imposed on visiting selected network vertices, the time when the UAV enters the next vertex $t_{jh}$ must be determined according to the Constraint (1). This means that flight times $T_{ij}$ between the vertices *i* and *j* of the network have to be taken into account. The recognition time of the object at the vertex $T_{i}^{I}$ must also be accounted for. In order to speed up the solver calculations in the drone swarm mission planning task, time windows $\left(e_{i},d_{i}\right)$ should be defined only for selected vertices (Constraints (2)–(3)). This only applies to those vertices that are assigned to the objects that need to be recognized (see [1,14]). The analyst should remember not to impose unnecessary time constraints. The time window is defined before planning the mission. It is given in the object recognition task. In practice, defining time windows is needed to identify priority targets that can only be recognized at a given time.

Constraint (1) includes the “big-M” component used for elimination of the logic part of this constraint. A short description of this method is presented in Appendix A.

The next three Constraints (4)–(6) model the possibility of recognizing the object by one or more UAVs, which will conduct reconnaissance flights along different segments of the designated routes. In the case of reconnaissance conducted by several platforms, this is a common situation. γ is the number of platforms that can perform recognition of the object. The model assumes that the analyst has defined only two such segments to identify one target (edges ((k,m)∈E,(l,n)∈E)). The presented constraints do not enforce the flight direction over the chosen edge.

The flight time of each UAV must not exceed its maximum capability denoted by $\tau_{h}$, which is modeled by the Constraint (7).

### 3.4. Constraints for Flow Networks

The flow network on which the optimization model is built enforces the definition of constraints that allow for determination of flight routes. These are the constraints specific to VRP class models. The constraints commonly used to model network flows and to eliminate subtours in VRP have been omitted. They may be checked in [1,14].

(8) $$\sum_{j\in V}y_{0jh}\leq 1\;,\;\forall \left(h\in H:q_{h}=1\right)$$

(9) $$\sum_{j\in V}y_{0jh}=1\;,\;\forall \left(h\in H:q_{h}=0\right)$$

(10) $$\sum_{j\in V}y_{jbh}=1\;,\;\forall \left(b\in V:b=V,h\in H\right)$$

(11) $$\sum_{h\in H}y_{ijh}\leq \kappa \;,\;\forall \left(i,j\in V:i\neq j\right)$$

This article assumes that the UAV working in a swarm as an information node must fly on each mission. The remaining platforms do not have to fly as long as only one UAV being the information node is equipped with the appropriate payload for observing the objects. Usually, there is no need for all platforms belonging to the swarm to start missions, which is described by the Constraints (8)–(9), where $q_{h}=0$ is the information node. Each UAV that has begun its mission must return to the base b∈V (10). The landing base number is equal to *V*. The take-off base is indexed 0. Constraint (11) ensures that each route segment can be visited more than once but less than κ. This is a modification introduced for the purposes of planning a swarm mission.

### 3.5. Constraints for the Swarm

The article presents two groups of constraints that relate to modeling the behavior of a swarm of drones. The first group of constraints concerns the planning of a swarm flight in such a way that UAVs are within communication range.

The second group of constraints concerns the modeling of the UAV’s flight plans on a network whose vertices model points at different heights above the ground level. This allows for better planning of the method of recognizing the terrain object using the sensor with which the UAV is equipped.

#### 3.5.1. Constraints for the Range of the Data Link

To define these constraints one must define the position of the ground control station that controls the swarm. GCS position is defined as ${\left(x,y,z\right)}^{GCS}$. Therefore, the constraint is

(12) $$\begin{array}{c} {||\left(x,y,z\right)}_{i},{\left(x,y,z\right)}^{GCS}\left|\right|\leq R+M\cdot \left(1-x_{ih}\right),\\ \forall (i\in V,h\in H:q_{h}=0) \end{array}$$

*R* means the safe distance from the GCS, where communication between the GCS and the UAV will be maintained even in unfavorable weather conditions. $q_{h}$ means that the UAV is an information hub that cooperates with the GCS and other UAVs. Equation (12) has a form of quadratic equation. A detailed algorithm for linearization of quadratic equations is presented in [14]. This article omits these transformations for the sake of simplicity.

(13) $$\begin{array}{c} \left(t_{ih}-t_{ju}\right)-\delta \leq M\cdot \left(1-z_{1ihju}\right),\\ \forall (i,j\in V),\forall (h,u\in H) \end{array}$$

(14) $$\begin{array}{c} \left(t_{ih}-t_{ju}\right)+\delta \geq -M\cdot \left(1-z_{2ihju}\right)\\ \forall (i,j\in V),\forall (h,u\in H) \end{array}$$

(15) $$\begin{array}{c} z_{1ihju}+z_{2ihju}\leq 2,\forall \left(i,j\in V\right),\forall \left(h,u\in H\right) \end{array}$$

(16) $$\begin{array}{c} {||\left(x,y,z\right)}_{i},{\left(x,y,z\right)}_{j}\left|\right|-R\leq M\cdot \left(1-x_{ih}\right)\\ +M\cdot \left(1-x_{ju}\right)+M\cdot \left(2-z_{1ihju}-z_{2ihju}\right),\\ \forall (i,j\in V),\forall (h,u\in H) \end{array}$$

Constraints (13)–(16) force each pair of unmanned platforms to be within communication range at any time during the flight. For this purpose, additional auxiliary *z* variables were introduced according to the big-M rule. The (16) constraint is only active when two UAVs occupy the corresponding vertices of the network at the same time, which is checked in Constraints (13)–(14). Vertices must be within a distance of *R*.

#### 3.5.2. Constraints for the Flight Altitude Restriction

The model assumes that the UAV can change the altitude in a given flight segment in accordance with its technical capabilities. Variable $z_{j}^{h}$ is the UAV’s flight altitude at vertex (waypoint) *j*. If the UAV flight altitude’s change is within the allowable range and the climb angle does not exceed the optimal β, the constraint that takes the form

(17) $$\left(z_{j}^{h}-z_{i}^{h}\right)/T_{ij}-\beta \leq 0$$

is transformed into

(18) $$\left(z_{j}^{h}-z_{i}^{h}\right)/T_{ij}-\beta \geq -M\cdot a_{1}$$

When the height is changed beyond the given angle $\beta_{max}$, the constraint

(19) $$\left(z_{j}^{h}-z_{i}^{h}\right)/T_{ij}-\beta_{max}\geq 0$$

is transformed into

(20) $$\beta_{max}-\left(z_{j}^{h}-z_{i}^{h}\right)/T_{ij}\geq -M\cdot a_{3}$$

If the change in UAV flight altitude is within the acceptable range, so the climb angle does not exceed the maximum angle of $\beta_{max}$, the constraints take the form

(21) $$\left(z_{j}^{h}-z_{i}^{h}\right)/T_{ij}-\beta \geq -M\cdot a_{2}$$

(22) $$\beta_{max}-\left(z_{j}^{h}-z_{i}^{h}\right)/T_{ij}\geq -M\cdot a_{2}$$

An additional equation must be satisfied:

(23) $$\sum_{i=1\ldots 3}a_{i}=1$$

In the described case, Constraint (1) can be written as

(24) $$T_{ij}=a_{1}\cdot T_{ij}^{opt}+a_{2}\cdot T_{ij}^{avg}+a_{3}\cdot T_{ij}^{max}$$

where $T_{ij}^{opt}$ represents the shortest flight time between the vertices *i* and *j*, $T_{ij}^{max}$ represents the longest flight time between the vertices *i* and *j* and $T_{ij}^{avg}$ represents the average flight time between the vertices *i* and *j*.

### 3.6. Optimization Objectives

Two optimization functions have been used in the presented model. The first function is used to maximize the profit obtained by a swarm visiting a set of route points, which increases with the number of objects identified:

(25) $$\sum_{i\in V,h\in H}\left(x_{ih}\cdot p_{i}\right)+\sum_{(i,j)\in V,h\in H}\left(y_{ijh}\cdot p_{ij}\right)$$

The objective function presented in (25) features two parts. The first one is used to sum a profit ($p_{i}$) from the visited vertices and the second part sums the profit from the visited arcs ($p_{ij}$). The profit value for each UAV that flies over the vertex of *i* or the edge of (i,j) is determined in this article as follows. If the object of interest to the analyst can be recognized from the vertex or edge, the value of $p_{i}$ or $p_{ij}$ is set to the maximum predefined value. The vertices that are in the vicinity of potential threats without the possibility of recognizing any object have the lowest value of profit. The same principle applies to determining the profit for the edge. It should be remembered, however, that it is the planner who decides to prioritize (profit from) the recognition of each element modeled by a vertex or an edge.

The second goal function used in the experiments minimizes the flight time of each UAV:

(26) $$\rho \sum_{i,j\in V}T_{ij}\cdot y_{ij}-\left[\sum_{i\in V}p_{i}\cdot x_{i}+\sum_{(i,j)\in E}p_{ij}\cdot y_{ij}\right]$$

This function features two parts. The first part with ρ as the optimization coefficient (ρ∈[0,1]) models the travel times of UAVs. The value of the coefficient reduces the impact of $T_{ij}$, which is the flight time of the drone between the vertices of the network. The next part models the profits from visiting some number of the targets with the predefined priorities. It is up to the analyst to determine whether to prefer the minimization of UAV flight time or prefer the maximization of recognized targets.

### 3.7. Route Replanning Scheme

An unmanned platform equipped with devices for recognizing and identifying dangerous objects must be equipped with algorithms that define how to avoid these threats. If it is necessary to plan an alternative route for the UAV after detecting a threat, it is possible to use one of the two approaches that fall into the category of local optimization problems, because the new route is defined for a short distance and, using these algorithms, it is not possible to calculate the entire plan from the beginning.

In [3], a route correction algorithm based on the dynamic domain rapidly-exploring random tree mechanism was proposed. This article takes into account both static and moving threats. In order for the algorithm to work properly, it is necessary to define the model of the flight dynamics of the unmanned platform.

For larger platforms, a different approach is used, as shown in [14]. When a threat is detected, the detection algorithm identifies the type of object that poses the threat. In this case, the site where the object is located is the center of the UAV’s prohibited area. The forbidden area is a hemisphere centered on the location of the threat. The UAV cannot enter this area. Based on the network on which the routes have been calculated, the algorithm of dynamic trajectory planning shown in [14] can be used and a method of avoiding the hazard can be determined.

An alternative to the described algorithms is the mechanism of correcting the flight paths of a swarm of drones based on recently determined routes. In such a case, MILP solvers provide a warm-start mechanism, which enables the task to be resolved and a global solution determined without the need to recalculate the entire task from the beginning. The solver remembers the necessary data that allows it to start computing in such a way that the last found solution is the initial solution of the new problem. In this case, if it is possible to correct the route (if such a route exists at all), the calculations will be much more efficient. The experiments carried out by the authors show that the route correction is usually determined in a time equal to 10–20% of the time needed to find the original flight paths of the swarm. The most important feature of this approach is that it does not differ from the solution of the basic task.

### 3.8. Object Detection with EO/IR

You Only Look Once (YOLO) is a real-time object detection algorithm that was created in 2016 by Joseph Redmon and Ali Farhadi [22]. In our approach, version 3 implemented in the ImageAI library is used [26]. The YOLO model has several advantages as compared to traditional methods of object detection and classification. Article [27] shows that YOLO can analyze 40–90 images per second. This means that streaming video can be processed in real-time, with negligible latency of a few milliseconds.

It encodes contextual information about classes and learns generalizable representations of items.

**Figure 3:**
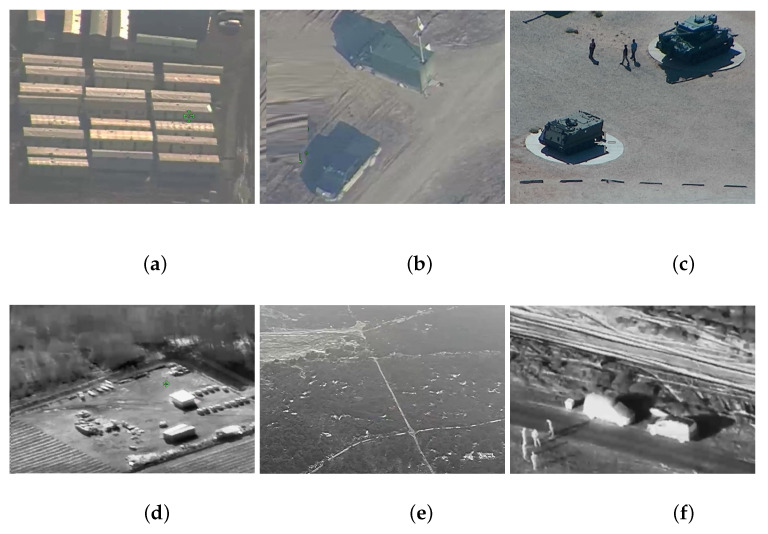
Sample images: (**a**–**c**) images obtained from an optoelectronic sensor; (**d**–**f**) images obtained from infrared sensor.

Figure 3 shows sample images obtained from an optoelectronic sensor (a–c) and an infrared camera (e–f).

**Figure 4:**
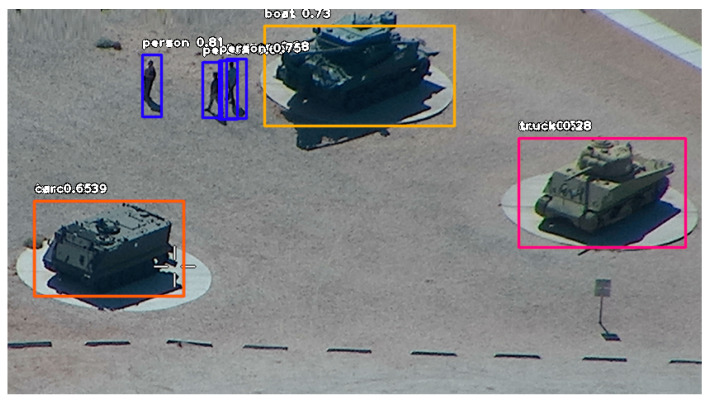
The result of image (Figure 3c) analysis.

Figure 4 shows the result of image analysis using the YOLOv3 network trained on the public database [26].

The image shows the location of the object, the name of the class and the probability of correct classification. The tanks are classified as boats or trucks. The misclassification is due to the fact that there were no images of tanks in the database. Objects in the images in Figure 3a and from the infrared camera were not recognized by the network. In Figure 3b, only the vehicle in the upper part of the image has been recognized.

Since there are no public databases containing images of military objects observed by aircraft, we started collecting a large dataset to teach the YOLOv3 network.

### 3.9. Object Detection with SAR

Synthetic aperture radar (SAR) is an imaging radar that is robust against different weather or light conditions [28] and provides high-resolution images representing the measure of the scene reflectivity [29]. A SAR system transmits an electromagnetic wave and receives an echo. The signal frequency ranges from hundreds of hertz for the airborne system to thousands of hertz for spaceborne approaches. In the case of the airborne system, SAR provides information on an area up to 400 km^2^. SAR images are usually affected by speckle noise [30,31] and distortion, so they are challenging to interpret.

**Figure 5:**
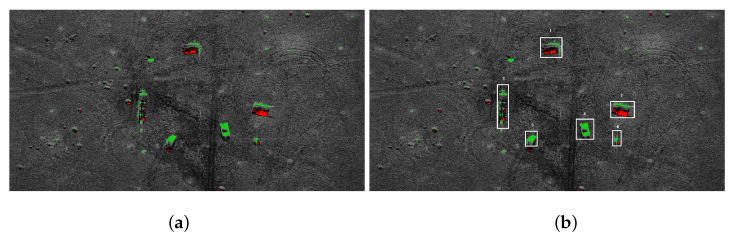
Extracted tanks: (**a**) shadow areas; (**b**) recognized objects.

We use the classic three-stage algorithm for dangerous object detection in SAR images. First, a median filter is applied to remove the noise [29]. A thresholding algorithm is used during segmentation [15]. Two kinds of region are extracted: shadow region (black pixels) and bright areas. Usually, shadows are adjacent to the underlying high objects, so they provide contextual information [32]. Then morphological closing [33] is applied to remove the noise. Figure 5a presents the results of the shadow extraction in a military training ground. Red pixels represent the shadow areas. The shadows are next to tanks and infantry fighting vehicles (the brightest spots in the image). The groups of bright pixels need further analysis.

In the case of tanks and trucks it is assumed that the extracted bright area is within 7 × 3 m. The expected size of the target surface is determined based on the pixel resolution in the SAR scan.

Shadows are not observed near small or short objects. Such objects require a different method of analysis. If vehicles are detected, we look for near-rectangular areas occupied by bright pixels. For a group of bright pixels the minimal area rectangle is computed. If the area of the rectangle is within the required range, we assume that the vehicle is recognized. Area no. 4 and 5 in Figure 5b have been recognized as vehicles; areas 3 and 6 are unclassified.

**Figure 6:**
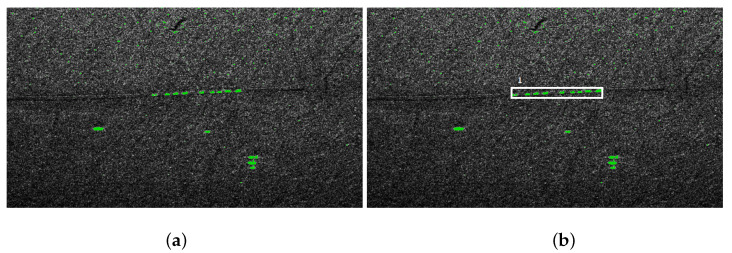
Convoy detection: (**a**) SAR image; (**b**) recognized convoy.

The group of areas recognized as vehicles can be analyzed and classified. We assume that the convoy is a group of collinear vehicles and the distance between successive objects is within certain limits. In Figure 6a, an image of a convoy is presented, Figure 6b presents extracted convoy of tanks.

In our experiments, a Jetson TX2 Tegra system-on-chip (SoC)-class computer was used. Low power consumption and high computing power are the main advantages of the platform, hence it is quite popular in UAV systems. Two kinds of power modes were tested:

- MAXQ—maximizing power efficiency—power budget up to 7.5 W, 1200 MHz Cortex A57 CPU, 850 MHz built-in Nvidia Pascal GPU with 256 CUDA cores,
- MAXN—maximizing processing power—power budget around 15 W, 2000 MHz Cortex A57 CPU, 2000 MHz Denver D15 CPU (not utilized in our tests) and 1300 MHz built-in GPU.

All algorithms were written and executed in C++ 14 standard.

Table 1 shows the average time of the convoy detection.

In Table 2 the time of object recognition is presented.

**Figure 7:**
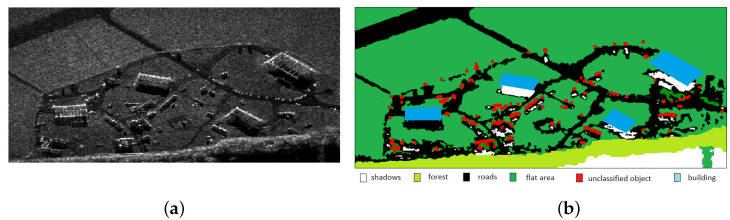
The result of image segmentation and classification: (**a**) SAR image; (**b**) segmented image [34].

In the article [34], the results of SAR image segmentation and area classification are presented (Figure 7). This kind of information can improve the process of planning the aircraft’s mission and flight execution.

## 4. Results

### Mission Plan Construction

In the presented research, a terrain model in the form of a directed network was used, which was composed of several vertices. This article presents the results for a network consisting of 10, 20 and 30 vertices for a swarm of 3–5 UAVs. Mission planning was tested for 2 different optimization models presented in Table 3.

Mission plans have been designated for cases where all the targets could be observed by the UAV from an optimal flight altitude (see Table 4). The calculations were stopped when the solver found an optimal solution or a suboptimal solution close to the optimal one (MILP Gap less than 10% in CPLEX solver). In the second case, the computation was terminated when, for a specified period of time, the solver did not find a better solution than the best one so far.

In each optimization task, six vertices that the UAVs must visit were determined (the vertices with the highest priorities). These vertices were used to model the recognition sites of the most important objects. Mission plans were designated for cases where one or more targets could only be observed by the UAV from a height greater than the optimal flight altitude.

The times given in Table 4 refer to the near-optimal solution, where the distance from the optimal solution is given in the Gap MILP column. Characteristic for VRPTW tasks is that an acceptable solution close to the optimal one can be found quickly, but finding the optimal solution at a given Gap MILP value may take a long time. Therefore, it is usually not worth waiting for such a solution and one should use a suboptimal solution.

**Figure 8:**
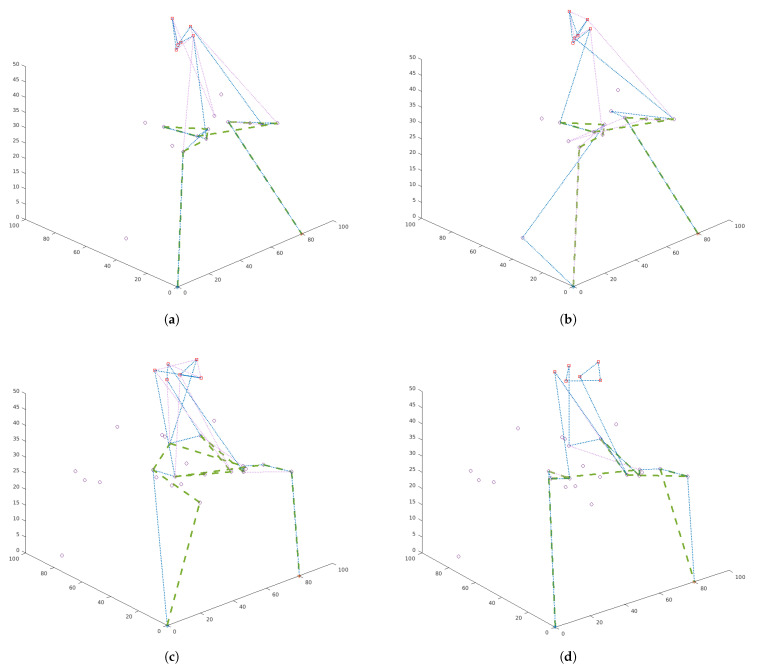
Route plans for a swarm with three UAVs: (**a**) network with 20 vertices, model I (total profit maximization); (**b**) network with 30 nodes, model II (minimization of the route length); (**c**) network of 30 vertices, model I; (**d**) network with 30 vertices, model I.

One should pay attention to the route plan for the swarm shown in Figure 8 for the presented cases. In Figure 8a,c the swarm recognizes objects as long as the capabilities of the platforms allow it (in this case the maximum flight times). Therefore, UAVs belonging to the swarm fly through the most important vertices marked in red several times (depending on the value of γ parameter). In Figure 8b,d the platforms belonging to the swarm are in the air until all of the most important objects are checked. Therefore, UAVs belonging to the swarm fly through the most important vertices marked in red not more than once. Depending on the task that will be set for the swarm, the swarm can be sent for single or multiple recognition of the object. Multiple flights in the neighborhood of the recognized object give greater certainty of recognition, but increase the risk of losing platforms.

## 5. Conclusions and Future Works

A framework for planning and execution of drone swarm missions in a hostile environment was presented. Two main components were described: the methods of planning drone swarm routes and the methods of detecting potentially dangerous objects in photos from EO/IR and SAR. The method of using data from the image analysis performed automatically by the UAV was also presented. The ways of replanning the flight paths of a swarm of drones were discussed. The mathematical model for the purpose of determining the swarm’s flight routes was given based on the example of a swarm with one UAV being an information hub. Such a model is used in reality and is becoming more and more popular, especially in military applications.

The time of operation of the exact algorithm does not prevent its practical application in a GCS. Even medium-sized GCSs have efficient computing environments that can be used to solve optimization tasks, so there is little need to use approximate algorithms. The use of approximate algorithms in practice is of course possible, but this topic was not discussed in the article due to the large number of works available in the literature. None of these articles, however, referred to the model of swarm operation presented in this article.

The algorithms for detecting objects in the terrain using SAR scans, which are collected by UAVs that recognize the indicated area, were presented. Each of the described algorithms was implemented on the Jetson Tegra X2i mobile platform.

The main contribution to the development of research on image analysis is to show the method of using detection and classification algorithms on lightweight mobile platforms that can be installed on UAVs.

The presented algorithms are widely used in tasks related to the analysis of UAV speed in the case of GPS errors. Image analysis can be used to determine the speed of the UAV and supports the inertial navigation system to accurately determine the geolocation of the UAV.

In our future works, we will investigate the ways to modify the mission if dangerous objects (tanks, vehicles) are detected. The segmented images (obtained from the drone’s sensors) will be compared to digital maps, making it possible to determine the drones’ location, even if a GPS signal is not available. The time of such image analysis is essential during the mission execution. The experimental results confirm that the stream of images can be analyzed in real-time.

## Figures and Tables

**Table 1:** Convoy classification time.

Jetson Tegra TX2	MAXQ	35 ms
	MAXN	20 ms
Personal Computer		7.5 ms

**Table 2:** Object recognition.

Jetson Tegra TX2	MAXQ	69.1 ms
	MAXN	40.08 ms
Personal Computer		11.9 ms

**Table 3:** Optimization models tested.

Model	Optimization Function	Constraints
I	max (25)	(1)–(7), (8)–(11), (12)–(16), (17)–(24)
II	min (26)	(1)–(7), (8)–(11), (12)–(16), (17)–(24)

**Table 4:** Times for solving an optimization problem by CPLEX for the predefined targets. The times given are the minimum times for the solver to find the best feasible solution in the shortest possible time. The calculations were made on a platform with an i5 processor with CPLEX ver. 20.

Model	No of	Point	Linear	κ	Min Time	Gap MILP
	UAVs	Targets	Targets		(s)	(%)
I	3	10	2	1	<1	0.1
II	3	10	2	1	<1	0.1
I	3	20	4	1	50	4
II	3	20	4	2	40	4
I	4	20	4	1	84	5
II	4	20	4	2	190	6
I	5	20	4	1	150	7
II	5	20	4	2	84	5
II	4	30	4	2	240	9
II	4	30	4	3	240	8
I	5	30	4	3	268	10
II	5	30	4	3	260	10

## Data Availability

The source code and data used to support the findings of this study are available from the corresponding author upon request.

## Abbreviations

The following abbreviations are used in this manuscript:

UAV	Unmanned air vehicle
GCS	Ground control station
VRP	Vehicle route planning
VRPTW	Vehicle route planning with time windows
MILP	Mixed integer linear programming
SAR	Synthetic aperture radar

## Appendix A

### Logics and Integer-Programming Representations

In mixed-integer linear models, the big-M rule is used with the related constant *M*. The big-M method transforms logical constraints into algebraic ones. The principle of the method is to bind the constraints to large negative constants that would not be part of any optimal solution.

The logical constraint representation b⇒f(x)≤0 is converted to

(A1) $$\begin{array}{c} f(x)\leq M\cdot (1-b) \end{array}$$

To ensure a constraint holds when a binary is true, we model the implication using a big-M strategy.

The logical constraint representation b⇒f(x)<0 is converted to

(A2) $$\begin{array}{c} f(x)\leq -\epsilon +M\cdot (1-b) \end{array}$$

To ensure a constraint holds when a binary is true, we model the implication using a big-M strategy.

The logical constraint representation f(x)≤0⇒b is converted to

(A3) $$\begin{array}{c} f(x)\geq -M\cdot b \end{array}$$

When f(x) becomes negative, the binary variable should be forced to be activated. Note that a non-strict inequality is used. If behavior around f(x) is important, a margin will have to be used as discussed before.

Representation of abs(x) as an integer programming constraint is as follows:

(A4) $$\begin{array}{c} x\geq -M\cdot (1-b_{1})\\ x\leq M\cdot (1-b_{2})\\ b_{1}+b_{2}=1 \end{array}$$

Representation of max(x) as an integer programming constraint is as follows:

(A5) $$\begin{array}{c} x-x_{1}\leq M\cdot \left(1-b_{1}\right)\\ \vdots \\ x-x_{n}\leq M\cdot \left(1-b_{N}\right)\\ \sum_{i=1\ldots N}b_{i}=1 \end{array}$$

## References

[1] Stecz W., Gromada K. (2020). UAV Mission Planning with SAR Application. Sensors.

[2] Wang L., Lu D., Zhang Y., Wang X. (2018). A Complex Network Theory-Based Modeling Framework for Unmanned Aerial Vehicle Swarms. Sensors.

[3] Wen N., Su X., Ma P. (2017). Online UAV path planning in uncertain and hostile environments. Int. J. Mach. Learn. Cyber..

[4] Boskovic J.D., Prasanth R., Mehra R.K. (2004). A multi-layer autonomous intelligent control architecture for unmanned aerial vehicles. J. Aerosp. Comput. Inf..

[5] Zhou Y., Rao B., Wang W. (2020). UAV Swarm Intelligence: Recent Advances and Future Trends. IEEE Access.

[6] Brand M., Masuda M., Wehner N., Yu X.H. Ant colony optimization algorithm for robot path planning. Proceedings of the 2010 International Conference on Computer Design and Applications.

[7] Butenko S., Murphey R., Pardalos P. (2006). Cooperative Control: Models, Application and Alogorithms.

[8] Karaboga D., Basturk B. (2008). On the performance of artificial bee colony (ABC) algorithm. Appl. Soft Comput..

[9] Luo R., Zheng H., Guo J. (2020). Solving the Multi-Functional Heterogeneous UAV Cooperative Mission Planning Problem Using Multi-Swarm Fruit Fly Optimization Algorithm. Sensors.

[10] Singgih I.K., Lee J., Kim B. (2020). Node and Edge Drone Surveillance Problem With Consideration of Required Observation Quality and Battery Replacement. IEEE Access.

[11] Xin J., Zhong J., Yang F., Cui Y., Sheng J. (2019). An Improved Genetic Algorithm for Path-Planning of Unmanned Surface Vehicle. Sensors.

[12] Liu T., Jiang Z., Geng N. (2013). A memetic algorithm with iterated local search for the capacitated arc routing problem. Int. J. Prod. Res..

[13] Chow J.Y.J. (2016). Dynamic UAV-based traffic monitoring under uncertainty as a stochastic arc-inventory routing policy. Int. J. Transp. Sci. Technol..

[14] Stecz W., Gromada K. (2020). Determining UAV Flight Trajectory for Target Recognition Using EO/IR and SAR. Sensors.

[15] Natteshan N.V.S., Suresh Kumar N. (2020). Effective SAR image segmentation and classification of crop areas using MRG and CDNN techniques. Eur. J. Remote Sens..

[16] Sezgin M., Sankur B. (2004). Survey over image thresholding techniques and quantitative performance evaluation. J. Electron. Imaging.

[17] Dalal N., Triggs B. Histograms of oriented gradients for human detection. Proceedings of the International Conference on Computer Vision & Pattern Recognition (CVPR’05).

[18] Xia C., Sun S.F., Chen P., Luo H., Dong F.M., Ooi W.T., Snoek C.G.M., Tan H.K., Ho C.K., Huet B., Ngo C.W. (2014). Haar-Like and HOG Fusion Based Object Tracking. Advances in Multimedia Information Processing—PCM 2014.

[19] Russell S., Norvig P. (2020). Artificial Intelligence: A Modern Approach.

[20] Hasni A., Hanifi M., Anibou C., Saidi M. (2020). Deep Learning for SAR Image Classification. Intelligent Systems and Applications.

[21] https://towardsdatascience.com/review-retinanet-focal-loss-object-detection-38fba6afabe4.

[22] Ahmad T., Ma Y., Yahya M., Ahmad B., Nazir S. (2020). Object Detection through Modified YOLO Neural Network. Sci. Program..

[23] https://arxiv.org/pdf/1504.08083.pdf.

[24] http://www.image-net.org/.

[25] https://www.cityscapes-dataset.com/.

[26] https://buildmedia.readthedocs.org/media/pdf/imageai/latest/imageai.pdf.

[27] Shinde S., Kothari A., Gupta V. (2018). YOLO based Human Action Recognition and Localization. Procedia Comput. Sci..

[28] Jung J., Yun S.H. (2015). Evaluation of Coherent and Incoherent Landslide, Detection Methods Based on Synthetic Aperture Radar for Rapid Response: A Case Study for the 2018 Hokkaido Landslides. Remote Sens..

[29] Manikandan S., Chhabi N., Vardhani J.P., Vengadarajan A. Gradient based Adaptive Median filter for removal of Speckle noise in Airborne Synthetic Aperture Radar Images. Proceedings of the International Conference on Signal, Image Processing and Applications with Workshop.

[30] Maryam M., Rajabi M., Blais J. (2006). Effects and Performance of Speckle Noise Reduction Filters on Active Radar and Sar Images. Proc. IRPRS.

[31] Zhu J., Wen J., Zhang Y. A new algorithm for SAR image despeckling using an enhanced Lee filter and median filter. Proceedings of the 6th International Congress on Image and Signal Processing (CISP).

[32] Borji A., Itti L. (2013). State-of-the-Art in Visual Attention Modeling. IEEE Trans. Pattern Anal. Mach. Intell..

[33] Poodanchi M., Akbarizadeh G., Sobhanifar E., Ansari-Asl K. SAR image segmentation using morphological thresholding. Proceedings of the 6th Conference on Information and Knowledge Technology (IKT).

[34] Siemiątkowska B., Gromada K. (2021). A New Approach to the Histogram Based Segmentation of Synthetic Aperture Radar Images. J. Autom. Mob. Robot. Intell. Syst..

